# Inflammatory diarrhea due to enteroaggregative *Escherichia coli*: evidence from clinical and mice model studies

**DOI:** 10.1186/1757-4749-5-36

**Published:** 2013-12-03

**Authors:** Dhira Rani Saha, Sucharita Guin, Rajendran Krishnan, Dhrubajyoti Nag, Hemanta Koley, Sumio Shinoda, Thandavarayan Ramamurthy

**Affiliations:** 1 Division of Histology & Electron microscopy, National Institute of Cholera and Enteric Diseases, P-33, C.I.T Road, Scheme XM, Beliaghata, Kolkata 700 010, West Bengal, India; 2 Division of Bacteriology, National Institute of Cholera and Enteric Diseases, P-33, C.I.T Road, Beliaghata, Kolkata 700 010, West Bengal, India; 3 Division of Data Management, National Institute of Cholera and Enteric Diseases, P-33, C.I.T Road, Beliaghata, Kolkata 700 010, West Bengal, India; 4 Collaborative Research Center of Okayama University for Infectious Diseases in India, P-33, C.I.T Road, Beliaghata, Kolkata 700 010, West Bengal, India

**Keywords:** Diarrhea, EAEC, Inflammation, Fecal leucocytes, Histology

## Abstract

**Background:**

This study was conducted to determine the role of enteroaggregative *Escherichia coli* (EAEC) in inflammatory diarrhea among hospitalized patients in Kolkata. The inflammatory pathogenesis of EAEC was established in mice model and histopathological studies. Presence of fecal leucocytes (FLCs) can be suspected for EAEC infection solely or as a mixed with other enteric pathogens.

**Methods:**

Active surveillance was conducted for 2 years on 2 random days per week with every 5^th^ patient admitted to the Infectious Diseases Hospital (IDH). Diarrheal samples were processed by conventional culture, microscopy, ELISA and molecular methods. Two EAEC isolated as sole pathogens were examined in mice after induced intestinal infection. The intestinal tissue samples were processed to analyze the histological changes.

**Results:**

Of the 2519 samples screened, fecal leucocytes, erythrocytes and occult blood were detected in 1629 samples. Most of the patients had acute watery diarrhea (75%) and vomiting (78%). *Vibrio cholerae* O1 was the main pathogen in patients of 5–10 years age group (33%). Shigellosis was more in children from 2–5 years of age (19%), whereas children <2 years appeared to be susceptible for infection caused by EAEC (16%). When tested for the pathogenicity, the EAEC strains colonized well and caused inflammatory infection in the gut mucosa of BALB/C mice.

**Conclusion:**

This hospital-based surveillance revealed prevalence of large number of inflammatory diarrhea. EAEC was the suspected pathogen and <2 years children appeared to be the most susceptible age group. BALB/C mice may be a suitable animal model to study the EAEC-mediated pathogenesis.

## Background

Gastroenteritis is a very common illness among children living in tropical regions. At the global level, it was estimated that about 1.87 million children aged less than 5 years die due to diarrhea [1]. Etiologically, a large number of bacteria, viruses and parasites are responsible in causing diarrheal illness. Among different pathogroups of diarrheagenic *Escherichia coli* (DEC), enteroaggregative *E. coli* (EAEC) is gaining importance as it causes persistent and acute diarrhea both in developing and developed countries [2,3]. In children, EAEC induced diarrhea is commonly associated with mucoid stool, which may sometimes contain blood and fecal leucocytes (FLCs) [4,5]. In USA, EAEC is the most common cause of diarrheal illness among all age groups [6]. Despite lots of information available on the virulence determinants of EAEC-mediated diarrhea, studies on the level of FLCs and associated histopathogenesis in gut mucosa are very less [7,8]. From diarrheal disease surveillance at the National Institute of Cholera and Enteric Diseases (NICED), Kolkata, we conducted a preliminary study to understand the role of EAEC in inflammatory diarrhea among hospitalized patients. We have identified involvement of EAEC as a sole pathogen in most of the cases and also with other enteric pathogens. Statistically, the FLC was significantly associated with the presence of EAEC along with the other pathogens (mixed infection). We also studied the inflammatory changes induced by EAEC in the mice model with two strains isolated as sole pathogens from the diarrheal patients.

## Results

Of the 2519 diarrheal stool specimens collected from November 2007 to October 2009, FLCs, erythrocytes and occult blood were detected in 1629 samples (65%). FLC was mainly detected with acute watery diarrhea (75%) and the rest (25%) of the patients had bloody (4.3%), loose stool (20.1%) and mucoid (0.5%) stool. Fever was recorded in 6% of the patients but the frequency of vomiting was high (78%). Age group difference of diarrheal cases with various enteric pathogens and FLCs are shown in (Table 1). Children <2 years seems susceptible to EAEC (16%), whereas in all age groups the EAEC infection rate was less (7%). Overall, the infection rate of *Vibrio cholerae* O1 and *Shigella* spp. were high in 5-10 years age group compared to EAEC.

**Table 1:** Age wise distribution of pathogens in FLC associated diarrheal patients at IDH, Kolkata

	**Age <1 yr (n=127)**	**1 to <5 yr (n=134)**	**2 to <5 yr (n=112)**	**5 to <10 yr (n=73)**	**10 to <18 yr (n=126)**	**Age <18 yr (n=1057)**	**All age Gr., (N=1629)**	**Ocult blood with diarrhoeal pathogens**	**Total samples processed (n=2519)**
	**n (%)**	**n (%)**	**n (%)**	**n (%)**	**n (%)**	**n (%)**	**n (%)**	**%**	**n (%)**
Bacteria									
*Vibrio cholerae* O1	14(11)	23(17.2)	28(25)	24(32.9)	42(33.3)	239(22.6)	370(22.7)	56.6	654(26)
*Vibrio cholerae* O139	0	0	0	1(1.4)	0	1(0.1)	2(0.1)	100	2(0.1)
*Vibrio cholerae* Non O1 Non O139	2(1.6)	0	0	4(5.5)	1(0.8)	32(3)	39(2.4)	70.9	55(2.2)
*V. parahaemolyticus*	1(0.8)	0	2(1.8)	41(5.5)	4(3.2)	61(5.8)	72(4.4)	97.3	74(2.9)
*Vibrio fluvialis*	3(2.4)	4(3)	1(0.9)	2(2.7)	2(1.6)	24(2.3)	36(2.2)	65.5	55(2.2)
*Aeromonas* spp.	0	1(0.7)	1(0.9)	1(1.4)	0	13(1.2)	16(1)	64	25(1)
*Campylobacter jejuni*	9(7.1)	18(13.4)	15(13.4)	4(5.5)	7(5.6)	34(3.2)	87(5.3)	73.7	118(4.7)
*E. coli*	1(0.8)	0	1(09)	0	4(3.2)	13(1.2)	19(1.2)	86.4	22(0.9)
*Shigellae*	6(4.7)	20(14.9)	21(18.8)	11(15.1)	10(7.9)	73(6.9)	141(8.7)	91.6	154(6.1)
*Salmonella*	0	1(0.7)	1(0.9)	1(1.4)	2(1.6)	14(1.3)	19(1.2)	82.6	23(0.9)
EPEC	8(6.3)	4(3)	2(1.8)	3(4.1)	0	18(1.7)	35(2.1)	77.8	45(1.8)
ETEC Group	3(2.4)	11(8.2)	3(2.7)	0	6(4.8)	61(5.8)	84(5.2)	73.7	114(4.5)
EAEC	20(15.7)	21(15.7)	11(9.8)	3(4.1)	8(6.3)	46(4.4)	109(6.7)	68.6	159(6.36)
Virus									
Rotavirus	64(50.4)	73(54.5)	28(25)	5(6.8)	10(7.9)	110(10.4)	290(17.8)	58.8	493(19.6)
Adenovirus	26(20.5)	18(13.4)	8(7.1)	3(4.1)	3(2.4)	32(3)	90(5.5)	71.4	126(5)
Norovirus G1	0	1(0.7)	0	0	0	3(0.3)	4(0.2)	66.7	6(0.2)
Norovirus G2	4(3.1)	6(4.5)	4(3.6)	1(1.4)	5(4)	16(1.5)	36(2.2)	50	72(2.9)
Sapovirus	6(4.7)	2(1.5)	4(3.6)	0	1(0.8)	13(1.2)	26(1.6)	63.4	41(1.6)
Astrovirus	3(2.4)	6(4.5)	4(3.6)	2(2.7)	4(3.2)	24(2.3)	43(2.6)	72.9	59(2.3)
Parasite		p							
*Blastocystis hominis*	0	0	0	1(1.4)	0	7(0.7)	8(0.5)	72.7	11(0.4)
*Entamaeba histolytica*	3(2.4)	10(7.5)	5(4.5)	1(1.4)	5(4)	45(4.3)	69(4.2)	84.1	82(3.3)
*Giardia lamblia*	14(11)	22(16.4)	20(17.9)	18(24.7)	25(19.8)	98(9.3)	197(12.1)	70.1	281(11.2)
*Cryptosporidium* spp.	22(17.3)	16(11.9)	10(8.9)	4(5.5)	4(3.2)	57(5.4)	113(6.9)	71.5	158(6.3)

**Figure 1:**
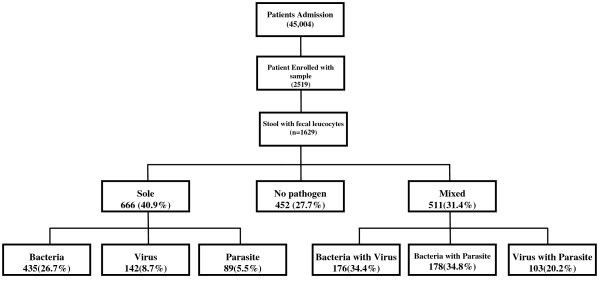
Flow diagram showing the description of sole and mixed pathogens detected among the acute diarrheal patients.

Diarrhea caused by a single pathogen with high FLC was detected in 666 cases (41%) than mixed pathogens 511 cases (31%)- Figure 1. EAEC was isolated as a sole pathogen in 23.9% of the cases and in 76.1% of the cases EAEC was identified as a mixed pathogen with other enteric bacteria/virus/parasite. Interestingly, FLC was significantly high when any of the *E. coli* pathogroups was present as a mixed pathogen (p = >0.001). Generally, high FLC has been detected in patients infected with enteric bacteria, which was 4 and 8 times high compared to parasites and enteric viral infections, respectively (Table 1).

**Figure 2:**
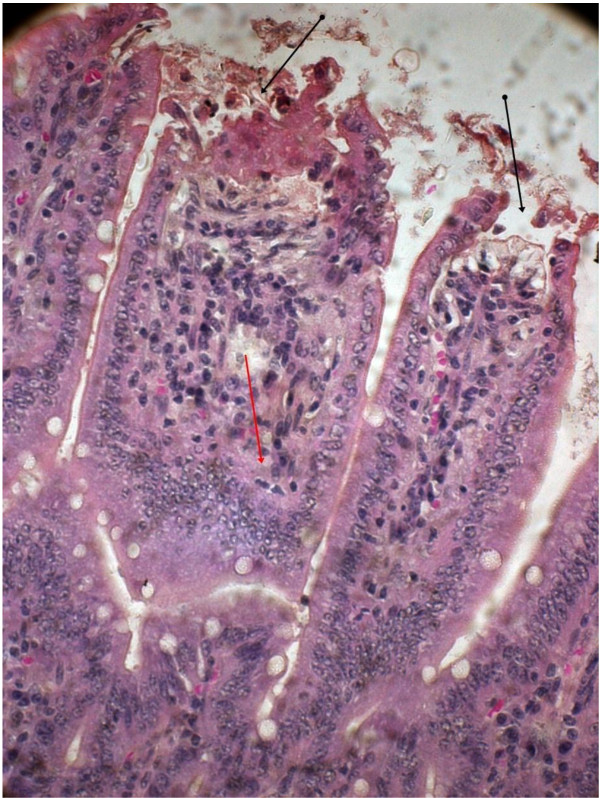
**EAEC strain 00611 treated mice intestinal mucosa on 10**^**th **^**day showing disrupted surface epithelium (black arrow) and exudates, grossly widened villous lamina propria with inflammatory cellular infiltrates (red arrow) (Hematoxylin & Eosin stain)-40x.**

**Figure 3:**
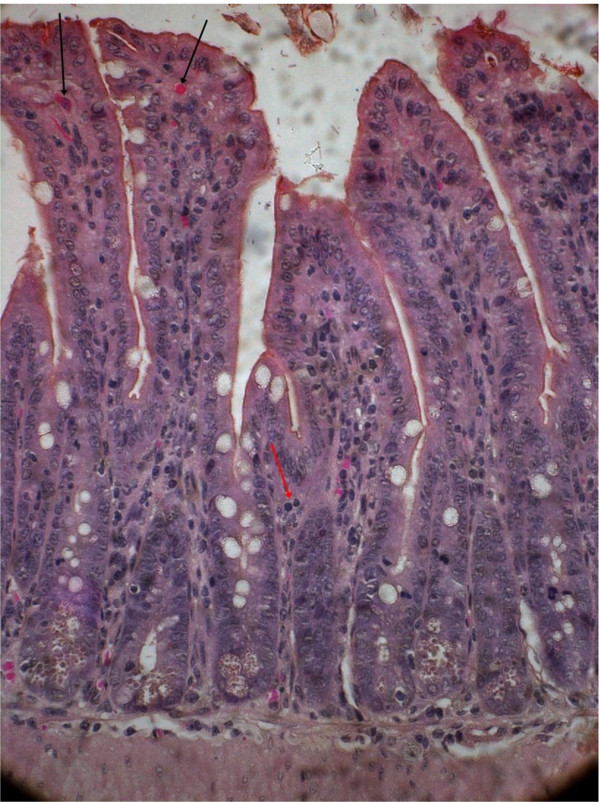
**EAEC strain 3544 treated mice intestinal mucosa on 10**^**th **^**day showing dilated villi with scattered hemorrhage (black arrow) and inflammatory cells (red arrow) (Hematoxylin & Eosin stain)-40x.**

**Figure 4:**
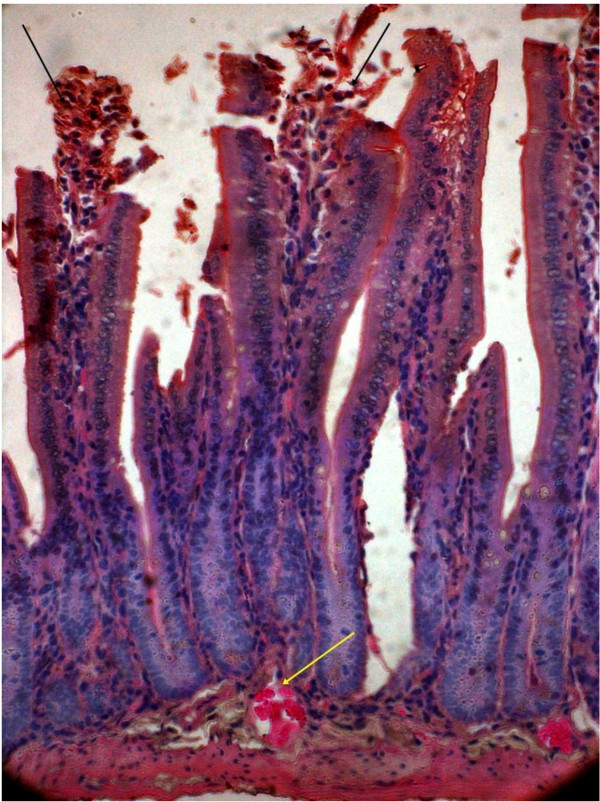
**Mice intestine on 11**^**th **^**day after 2**^**nd **^**challenge of 00611 strain showing grossly altered villous architecture, ruptured surface epithelium (black arrow), oedematous & congested lamina propria and submucosa (yellow arrow) with inflammatory cells (Hematoxylin & Eosin stain)-40x.**

**Figure 5:**
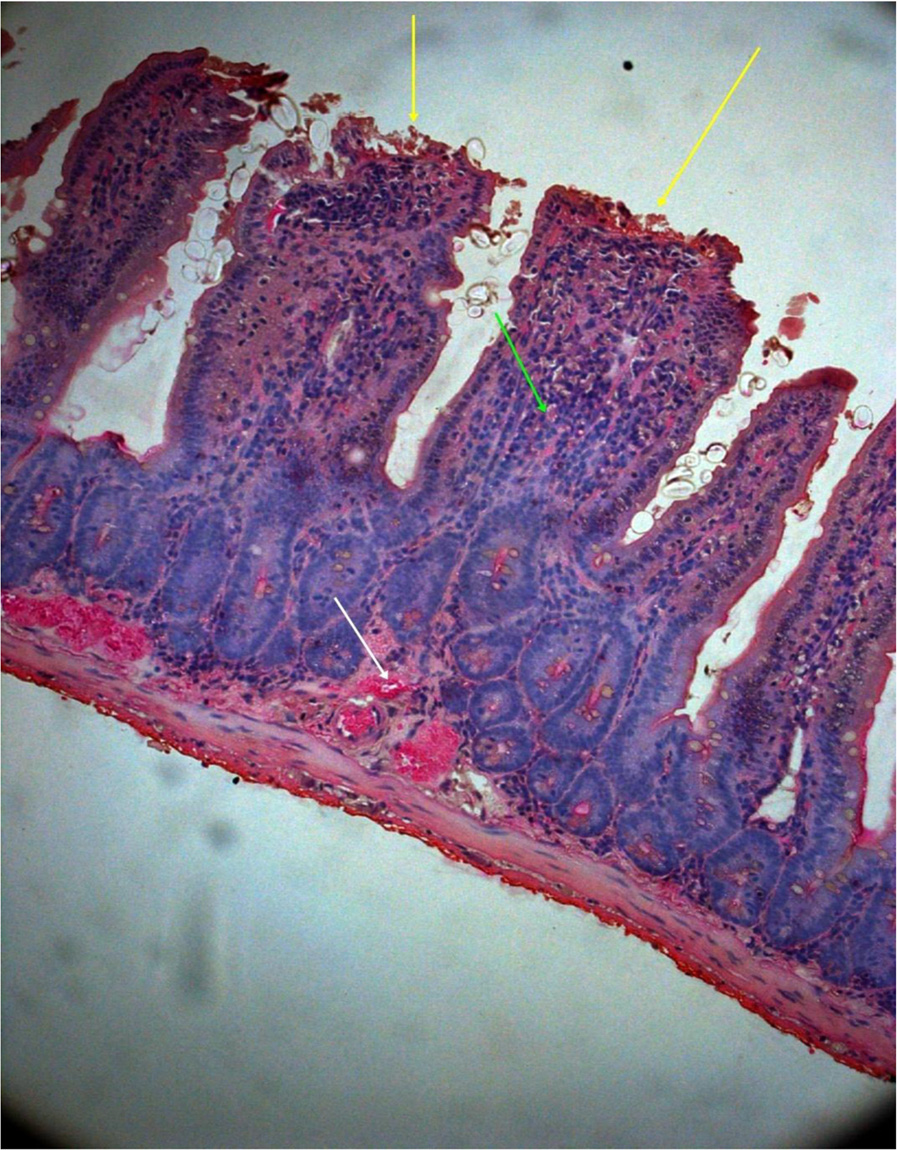
**Mice intestine on 11**^**th **^**day after 2**^**nd **^**challenge of 3544 strain showing damaged villous structure with widely dilated & congested lamina propria and submucosa (yellow arrow) and scattered inflammatory cells (green arrow) (Hematoxylin & Eosin stain)-40x.**

**Figure 6:**
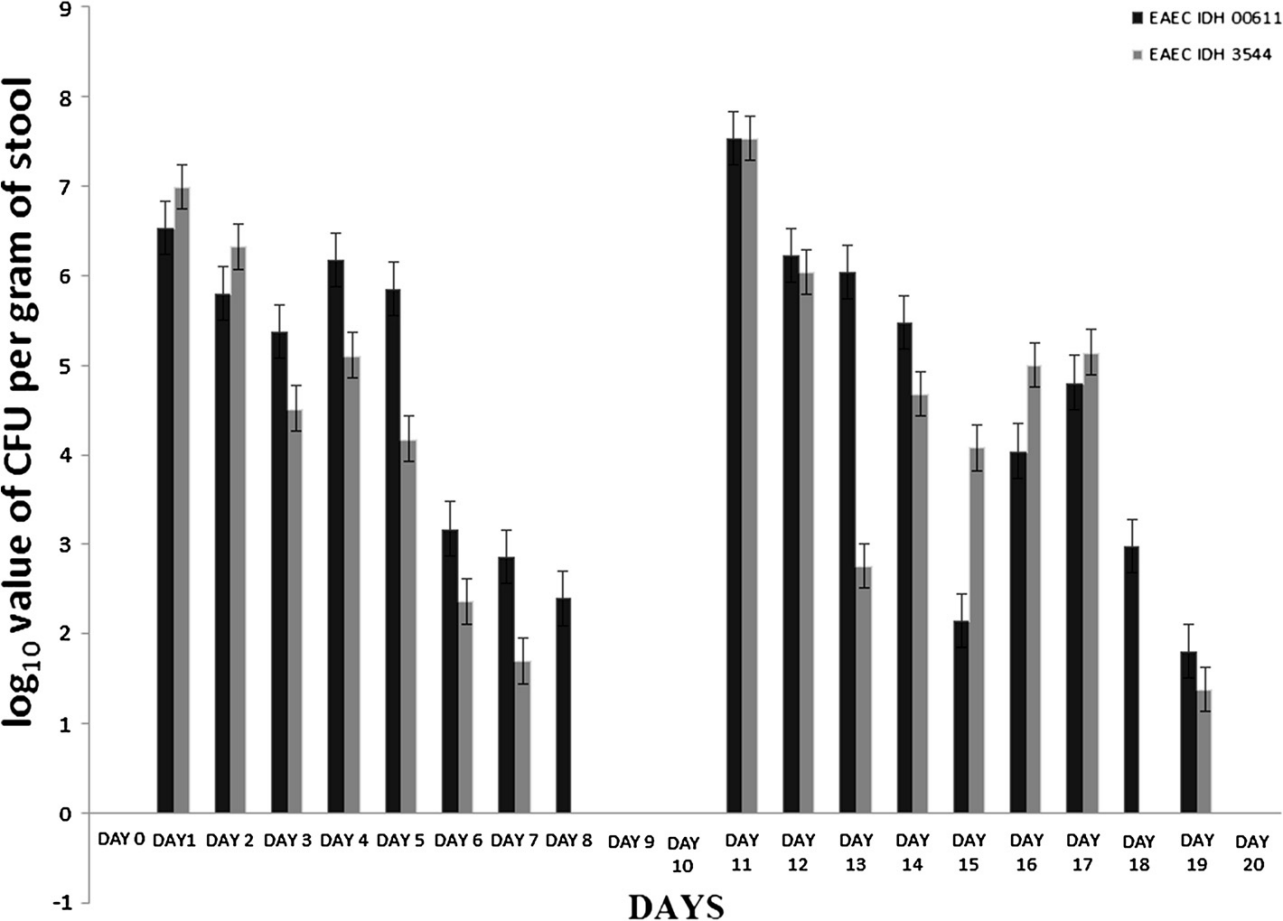
Day wise CFU count of bacterial shedding per gram of EAEC infected mice stool.

**Figure 7:**
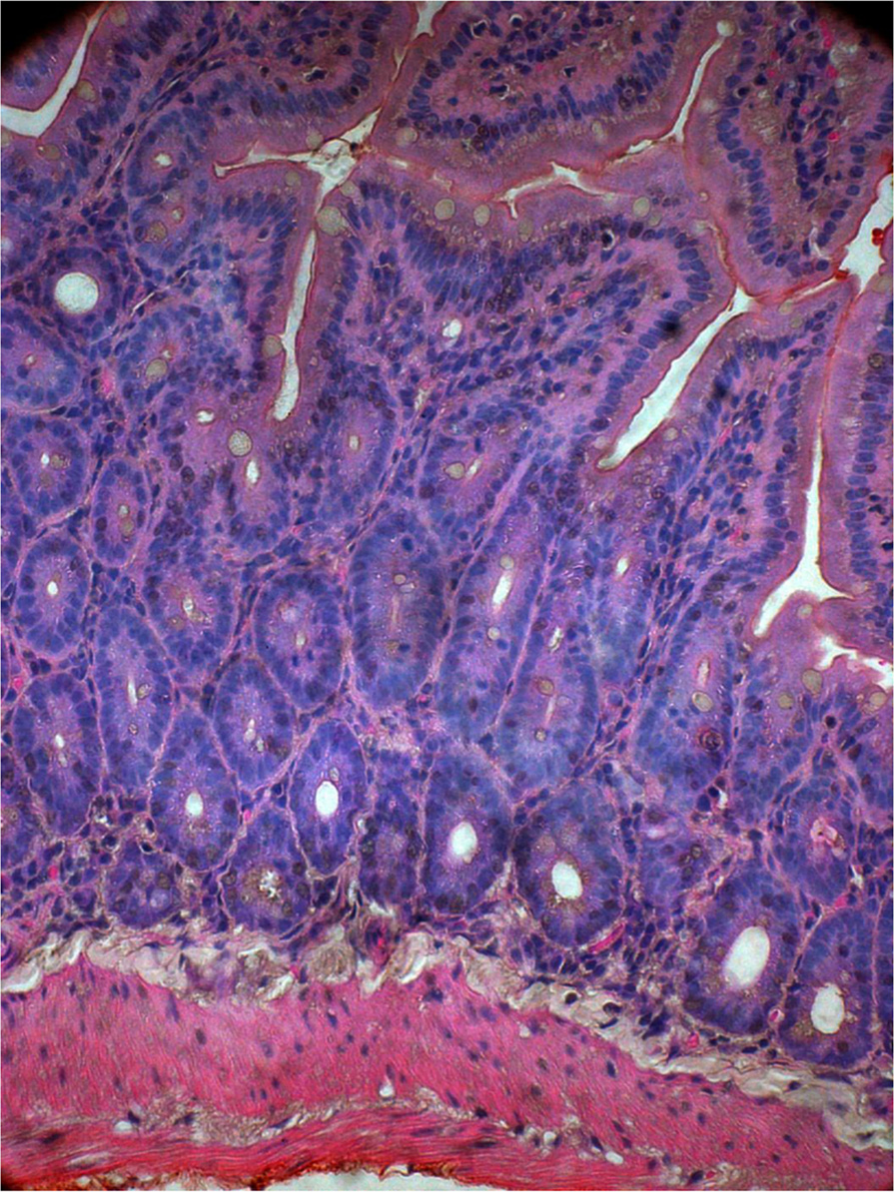
**Mice intestine almost coming back to normal on 20**^**th **^**day after 1**^**st **^**& 2**^**nd **^**challenge of EAEC strain 00611-40x.**

**Figure 8:**
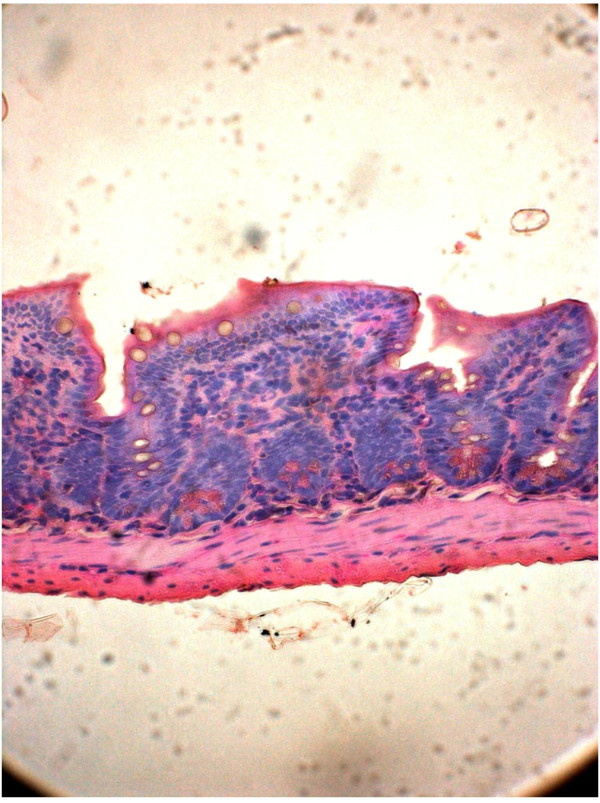
**Mice intestine treated with PBS on 10**^**th **^**day as control (Hematoxylin & Eosin stain)-40x.**

In order to understand the pathogenesis of EAEC, two serologically confirmed strains isolated as sole pathogen from diarrheal patients were tested in the BALB/C mice. On 10^th^ day of inoculation with the EAEC strain 00611 (Serogroup O6), ileum showed disrupted surface epithelium and exudates, grossly widened villous lamina propria with inflammatory cellular infiltrates basically consisting of mononuclear cells and neutrophils (Figure 2). Dilated villi with scattered hemorrhage and inflammatory cells were also observed with the other EAEC strain 3544 (serogroup O6) (Figure 3). After the 2^nd^ inoculation (10^th^ day from the 1^st^ dose), 4 animals from test and control groups were sacrificed on 11^th^ day. Histopathological study revealed gross alteration of villous architecture, damaged surface epithelium, oedematous and congested lamina propria and submucosa with inflammatory cells with both the EAEC strains. The hisopathological changes after 2^nd^ inoculation appeared more severe with both the strains (Figures 4 and 5). Bacterial shedding/gram of stool were maximum on 11^th^ day after 2^nd^ inoculation (Figure 6). After 1^st^ and 2^nd^ dose of infection, 3^rd^ set of sacrifice was made on 20^th^ day. Histomorphology of ileal tissue with each two strain showed almost normal appearance this time similar to that of control (Figure 7 and Figure 8).

## Discussion

Diarrhea and infectious enterocolitis is a major health problem especially among children in developing countries, where the child mortality rate has been high [9]. In this context, our two year surveillance study revealed inflammatory diarrhea in 65% of the cases, which is quite high. Since more than 20,000 acute diarrheal cases are getting admitted in the IDH per year, an active surveillance was made in which stool specimens were collected from every 5^th^ hospitalized patient with diarrhea or dysentery on two randomly selected days in a week.

FLCs in the diarrheal stool smear are suggestive of invasive etiology [10,11] and based on this estimation and fecal lactoferrin, inflammatory diarrhea can be determined. A wide range of pathogens in different age groups were identified in this study. Etiologically, bacterial diarrhea was the most predominant one. Children in the age group of 5 to >10 years seems more susceptible to *V. cholerae* infection (33%), whereas the age group up to 5 years were susceptible to shigellosis (19%). The younger age group children <2 years were susceptible to EAEC infection (16%).

Other than bacteria, viruses and parasites were also identified either as sole or in mixed pathogens. EAEC was identified with other enteric pathogens, which was statistically significant (p=>0.001). The duration of diarrhea among EAEC infected patients was long compared to other pathogens (data not shown). In many investigations, EAEC was found to be associated with persistent diarrhea [12,13].

In clinical settings, it must be challenging to distinguish inflammatory and non-inflammatory diarrhea. The results of our surveillance showed that acute watery diarrhea as the major clinical symptom in which 75% of the cases had FLCs and lactoferrin. This data indicates that inflammatory diarrhea is more in this region. In many investigations, the clinical presentation of EAEC infection was mostly associated with acute watery diarrhea especially among infants and young children with occasional presence of blood and mucus [14,15]. Considering identification of large number of acute diarrheal cases with the high FLCs, there is a need to correct dehydration in patients and reduce the infection of EAEC. This collective therapy may reduce the duration of stay in the hospital. Similar to our observation, other studies have also reported about the presence of FLCs with mild inflammation in DEC infected cases [16-19] especially by EAEC [20,21].

Disease manifestation and subsequent inflammatory changes were tested in mice model with human isolates of EAEC. As observed in many studies, oral challenge of the EAEC strains and subsequent bacterial shedding in the stools revealed prolonged colonization and significant inflammatory changes in intestinal tissue [22-24]. Histological sections of ileal tissue revealed damaged surface mucosa with inflammatory infiltrates in lamina propria spreading to the muscularis mucosa and submucosa. Maximum fecal shedding of the organism was noted after 2^nd^ oral inoculation on 11^th^ day and histopathological changes confirmed this observation.

Frank blood and pus in the stools were not observed in mice challenged with EAEC though loose stool with mucus was occasionally seen. Fecal shedding of the organisms and histological changes support mild inflammation in the mice infection studies. After 20 days from the 2^nd^ inoculation, no inflammation was noticed in mice. This indicates the normal recovery of animals in due course of time. However, this result cannot be correlated with human infection mediated by EAEC as the severity of infection is more pronounced. Experimental evidence showed that there may be an inhibition of water absorption due to damage of gut surface epithelium caused by enteric bacteria or its toxins [25]. Considering this aspect, the treatment of inflammatory diarrheal patients with oral rehydration solution may be less effective.

Most of the rural hospitals in India and other developing countries are not equipped with modern laboratory facilities for the early identification of pathogens from clinical specimens. In such settings, direct-microscopy will be supportive with other clinical finding and this approach may help the physicians to take prompt decision regarding management of acute diarrhea while expecting the diagnostic results. Screening of FLC is simple, rapid and cost effective and that can be done in outpatient department. ELISA and PCR techniques may give rapid results which are highly specific and sensitive but the conventional microscopy may act as a supportive tool for the simple diagnosis.

Our data suggest i) high FLC is frequently associated with bacterial diarrheas, ii) fecal leucocytes is 4 times higher in bacterial diarrhea than parasitic diarrhea and 8 times higher than viral diarrhea, iii) in the case of mixed infection, diarrheagenic *E. coli* may be the other suspected pathogen, iv) bacterial invasion in intestinal mucosa is a multistep process and in this regard, BALB/C mice could be an useful model to study the EAEC infection.

## Conclusions

This hospital based surveillance revealed high incidence of inflammatory diarrhea in Kolkata. Among the inflammatory diarrheal cases, the role of EAEC along with other pathogen(s) was found to be significant. This association was more pronounced in children >2 years of age. Histopathological studies with EAEC have shown typical inflammation in the gut microvilli. The BALB/C mice could be considered as an effective model for further study of EAEC pathogenesis.

## Methods

### Collection and analysis of stool specimens

From November 2007 to October 2009, a systematic active surveillance was conducted at the NICED to detect the prevalence of different enteric pathogens among acute diarrheal patients admitted at the IDH. Stool specimens were collected from all age groups from every 5^th^ hospitalized patient with diarrhea or dysentery on two randomly selected days (48 hrs) in a week. Two thousand five hundred nineteen stool specimens were collected in sterile containers and sent to the laboratory within 2 hrs. For the detection of enteric pathogens, conventional culture methods, immunologic and molecular microbiologic techniques were employed in the study [26].

### Fecal leucocytes and RBC in stool

As a marker of inflammation, FLC and red blood cells (RBC) were examined microscopically (Olympus CX41, Olympus Corporation, Tokyo, Japan) by smearing a thin layer of fresh stool on a glass slide after stained with methylene blue [27]. Stool specimen with FLCs >10 polymorphonuclear leucocytes in five or more fields under high power was considered positive.

### Fecal lactoferrin

The specimens positive for FLCs were further tested to determine the presence of fecal lactoferrin using an immunochromatographic detection kit (Leukoez value; Tech Lab, VA).

### Analysis of fecal occult blood (FOBT)

Microscopic presence of RBC was further confirmed by Hemaoccult 11 (Smithkline Diagnostics, San Jose, CA) [28,29].

### Selection of bacterial strain

Two EAEC strains 00611 and 3544, isolated as sole pathogens from diarrheal patients and belonging to serogroups O6 and O6, respectively were selected for further study along with a non-pathogenic *E. coli* strain DH5α. The EAEC strains were chloramphenicol resistant and grown in tryptic soy agar (TSA, Dfico, BD, Sparks, MD) spread plate method in four different concentrations of the antibiotic i.e., 25 μg/ml, 50 μg/ml, 75 μg/ml and 100 μg/ml. The EAEC strains were resistant to all the concentrations of chloramphenicol but the control strain (DH5α) remained susceptible for this antibiotic.

### Animal studies

The animal model study in this report was approved by the Institutional animal Ethical Committee (Apro/77/24/11/2010,Reg.No.NICED/CPCSEA(AW)215/2009-2015). Six weeks old male BALB/C mice were used in the study and were provided with sterilized water and food before and during the experiment. All the animals were kept in the germ-free environment. Twenty mice were inoculated intragastrically with two EAEC strains (ten in each group) (0.2 ml) by using a 1 ml tuberculin syringe fitted with a 20 gauge needle. The mice were closely observed to ensure that they did not regurgitate or aspirate the given inoculum. Ten mice were inoculated separately with 5% (w/v) filter sterilized phosphate buffered saline (0.2 ml) and used as control. Mice were closely monitored and stool samples were collected daily for bacteriological analysis. On 10^th^ day, 4 mice each from the test and control groups were sacrificed. The rest 12 mice in two test groups were reinoculated with the same dose of each of the EAEC strain and maintained under the same condition as mentioned before. On 11^th^ day after 2^nd^ inoculation, 2 mice from test group and 2 from control group were sacrificed. Mice feces were screened for the presence of the test strains over a period of 20 days and the last sacrifice of 4 test mice and 4 control mice was made on 20^th^ day of the experiment. After each batch of the experiment, intestinal tissue samples were preserved for histopathology. For specific enumeration and identification of EAEC, samples of freshly passed feces were homogenized in sterile phosphate-buffered saline (PBS), spread on TSA containing 75 μg of chloramphenicol, and incubated overnight at 37°C. The identity of the colonies resembling *E. coli* was confirmed by typical morphology and agglutination with the respective O serogroup rabbit antisera (Denka Seiken, Tokyo, Japan). The experiment was repeated two times and average of the test and control groups were recorded.

### Histology

Samples from mice intestine were fixed in 10% buffered formalin (pH 7.4), dehydrated and embedded in paraffin. Serial thin sections (3-5 μm) were made by Rotary microtome (Leica 2145, Germany) and stained with hematoxylin and eosin stain to observe the pathomorphological changes.

## Competing interests

The authors declare that they have no competing interests.

## Authors’ contributions

DRS conceived of the study and wrote the manuscript, TR, SS and HK designed the research SG and DN performed the research RK, statistically analysed the data. All authors read and approved the final manuscript.
